# Refinement of cryo-EM 3D maps with a self-supervised denoising model: *crefDenoiser*

**DOI:** 10.1107/S2052252524005918

**Published:** 2024-07-29

**Authors:** Ishaant Agarwal, Joanna Kaczmar-Michalska, Simon F. Nørrelykke, Andrzej J. Rzepiela

**Affiliations:** a Scientific Center for Optical and Electron Microscopy, ETH Zürich, 8093Zürich, Switzerland; b Department of Computer Science, Wrocław University of Science and Technology, 50-370Wrocław, Poland; c Department of Systems Biology Harvard Medical School,Boston MA02115 USA; Chinese Academy of Sciences, China

**Keywords:** 3D reconstruction and image processing, computational modeling, cryo-EM, denoising, neural networks

## Abstract

Cryogenic electron microscopy (cryo-EM) is a pivotal technique for imaging macromolecular structures. However, despite extensive processing of large image sets collected in cryo-EM experiments to amplify the signal-to-noise ratio, the reconstructed 3D protein-density maps are often limited in quality due to residual noise, which in turn affects the accuracy of the macromolecular representation. Here, *crefDenoiser* is introduced, a denoising neural network model designed to enhance the signal in 3D cryo-EM maps produced with standard processing pipelines. The *crefDenoiser* model is trained without the need for ‘clean’ ground-truth target maps. Instead, a custom dataset is employed, composed of real noisy protein half-maps sourced from the Electron Microscopy Data Bank repository. Competing with the current state-of-the-art, *crefDenoiser* is designed to optimize for the theoretical noise-free map during self-supervised training. We demonstrate that our model successfully amplifies the signal across a wide variety of protein maps, outperforming a classic map denoiser and following a network-based sharpening model. Without biasing the map, the proposed denoising method leads to improved visibility of protein structural features, including protein domains, secondary structure elements and modest high-resolution feature restoration.

## Abstract 2

State-of-the-art 3D cryo-EM map denoising with a self-supervised neural network model optimized for theoretical noise-free maps is introduced.

## 1. Introduction

### 1.1. Noise sources and denoising in cryo-EM

Cryogenic electron microscopy (cryo-EM) is one of the leading methods to elucidate protein structures (Bai *et al.*, 2015 ▸; Cheng, 2018 ▸). In a cryo-EM experiment, a low-intensity electron beam must be used in order to minimize the organic sample degradation during imaging, resulting in noisy images. Thousands of these noisy images are collected and processed to improve the low, significantly below 1 (Frank & Al-Ali, 1975 ▸; Egelman, 2016 ▸), signal-to-noise ratio (SNR). This approach ultimately allows for modeling detailed atomic resolution 3D protein maps (Nakane *et al.*, 2020 ▸). Still, the remaining noise is one of the factors limiting the reconstructed map’s quality (Rosenthal & Henderson, 2003 ▸; Frangakis, 2021 ▸).

The low-intensity electron beam is responsible for shot noise in the cryo-EM images. Furthermore, the protein particle projections are modulated by structural noise. This noise appears due to the non-uniform surroundings of the imaged particles: for example, amorphous ice impurities and ice thickness fluctuations. The resulting 3D protein-density maps are also affected by errors in data processing: for instance, inaccuracies of 3D image alignment (Jiménez-Moreno *et al.*, 2021 ▸).

The most successful cryo-EM denoising method so far is simply processing and averaging a large number of images. The improvement of the reconstructed map as a function of the number of acquired images can be estimated (Rosenthal & Henderson, 2003 ▸). The relation is logarithmic, which sets improvement limits due to acquisition costs. Other methods, such as 2D micrograph denoising (Palovcak *et al.*, 2020 ▸; Bepler *et al.*, 2020 ▸), 3D map denoising (Ramlaul *et al.*, 2019 ▸; Tegunov *et al.*, 2021 ▸) and corrections for 3D image alignment (Jiménez-Moreno *et al.*, 2021 ▸), are an active area of development and testing.

### 1.2. Deep learning enhances cryo-EM data processing

Deep learning has found extensive use in image processing. Therefore, the adoption of neural network models in cryo-EM processing is broad and the application of new methods has often been straightforward (Chung *et al.*, 2022 ▸). For example, the popular YOLO object detection network (Redmon *et al.*, 2016 ▸) was adapted to pick protein particles from EM images as *crYOLO* (Wagner *et al.*, 2019 ▸). Denoising network models, developed for general-purpose image denoising and restoration, can also be used for contrast enhancement in 2D EM images (Bepler *et al.*, 2020 ▸; Lehtinen *et al.*, 2018 ▸; Batson & Royer, 2019 ▸). There are also a number of specialized methods for 3D model building [*e.g.CryoDRGN* (Zhong *et al.*, 2021 ▸), *3DFlex* (Punjani & Fleet, 2023 ▸), GMM-based methods (Chen *et al.*, 2024 ▸)] map post-processing [*DeepEMhancer* (Sanchez-Garcia *et al.*, 2021 ▸), *EMReady* (He *et al.*, 2023 ▸)], map analysis [*DeepRes* (Ramírez-Aportela *et al.*, 2019 ▸)] and atomistic model building [*Emap2sec* (Maddhuri Venkata Subramaniya *et al.*, 2019 ▸), *ModelAngelo* (Jamali *et al.*, 2022 ▸)], which are powered by neural networks.

### 1.3. 3D map sharpening and denoising

Cryo-EM 3D density maps show a loss of contrast at high resolutions. This is caused by the decay of high-frequency signal amplitudes, which are smaller than expected when compared with the reference X-ray scattering data (Rosenthal & Henderson, 2003 ▸). Contrast degradation is caused by imperfect imaging due to inherent instrument limitations in transmission electron microscopy (TEM) apparatus, including specimen movement and charging, radiation damage, inelastic electron scattering events, partial microscope coherence, and particle flexibility and heterogeneity, and also due to the limitations of data-processing methods (Henderson, 1992 ▸; Rosenthal & Henderson, 2003 ▸). To restore the degraded signal, global sharpening methods and, more recently, local sharpening methods have been developed. *LocScale* (Jakobi *et al.*, 2017 ▸) uses an atomic reference structure to locally correct signal amplitudes. *LocalDeblur* (Ramírez-Aportela *et al.*, 2020 ▸) performs deblurring based on the local resolution estimation. *LocSpiral* (Kaur *et al.*, 2021 ▸) uses the spiral phase transform to enhance high-resolution map features. Vargas *et al.* (2022 ▸) utilize a multiscale tubular filter to enhance post-processed maps. *DeepEMhancer* (Sanchez-Garcia *et al.*, 2021 ▸) is a network model trained on pairs of raw experimental and sharpened maps. It uses *LocScale* (Jakobi *et al.*, 2017 ▸) maps as targets to mimic *LocScale*’s local sharpening effect without the need for atomic reference structures. *EMReady* is a similar method (He *et al.*, 2023 ▸), but trained on pairs of raw experimental maps and maps simulated from atomistic models. *EMReady* is optimized to match the ground-truth final post-processed maps but not necessarily to represent the raw experimental data optimally. Another widely used sharpening method is *phenix.auto_sharpen* (Terwilliger *et al.*, 2018 ▸), which has also been employed in this article to visually compare maps (see Section 2.6). Another relevant method, *LAFTER* (Ramlaul *et al.*, 2019 ▸), is a classic local 3D map denoising algorithm based on two serial filters. *LAFTER* operates in both real and Fourier space. It compares independent half-set reconstructions to identify and retain shared features with power greater than the noise. *LAFTER* does not sharpen EM maps, it only denoises them, which makes it a suitable reference method for benchmarking our network-based denoising model.

### 1.4. Contributions

Entries in the Electron Microscopy Data Bank (EMDB) repository contain not only the final processed cryo-EM 3D maps but also, in many cases, ‘half maps’, which result from processing two randomly divided half-datasets. The two half-maps are used for the determination of the map resolution (Van Hell & Schatz, 2005 ▸; Rosenthal & Henderson, 2003 ▸), and can be used to illustrate how the map’s SNR changes as a function of the signal frequency with Fourier shell correlation plots [FSC (Van Heel, 1987 ▸)] or by directly calculating power spectra of signal and noise components (Palovcak *et al.*, 2020 ▸). This type of data is suitable for training a neural network 3D map denoising model. The most natural setup would be the so-called noise-to-noise model (Lehtinen *et al.*, 2018 ▸), in which the first half-map is used as a denoising template and the second serves to calculate loss during the supervised model training. This is how the *M* software (Tegunov *et al.*, 2021 ▸) is, on the fly, training a map-specific model during a map refinement procedure. Here, we take advantage of existing theoretical analysis to further enhance the model’s denoising power. Rosenthal & Henderson (2003 ▸) derive a relation between an ideal noise-free 3D map and a pair of two noisy half-maps as a function of FSC. In *Methods*[Sec sec2], we outline how we employ this relation to optimize the denoising network in self-supervised training. We compare our model *crefDenoiser* with the recent 3D map denoiser *LAFTER* (Ramlaul *et al.*, 2019 ▸), the sharpening model *EMReady* (He *et al.*, 2023 ▸) and the pre-trained 3D *Topaz* denoising model (Bepler *et al.*, 2020 ▸; specific for cryo-electron tomography data, *Topaz*^*Tomo*^), and analyze their denoising performance on the test maps set with a number of selected characteristics. Furthermore, we analyze the signal-to-noise enhancements as a function of signal frequency, and we show that *crefDenoiser* improves the SNR, without introducing large biases in the denoised maps. This is in contrast to the *EMReady* model, whose primary role is to enhance maps by ingesting additional signal to the maps, rather than filtering the noise.

Finally, we provide examples of denoising with selected maps, where our processing provides insights into the usability and advantages of denoising.

## 2. Methods

### 2.1. Model optimization

The *crefDenoiser* model is trained using a loss function based on FSC and the statistical measure known as *C*_ref_. FSC score is the most popular metric used in cryo-EM imaging to determine image and map quality (Rosenthal & Henderson, 2003 ▸). It measures the normalized cross-correlation between two volumes over corresponding shells in the Fourier domain. It quantifies the similarity of signals between two maps (or images when a 2D signal is analyzed) as a function of frequency. The FSC value between two map volumes is given by
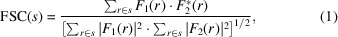
where *F*_1_ and 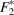 represent the Fourier transform and conjugate Fourier transform of the two volumes, and *s* is the shell being considered. The summation is performed over all frequency voxels *r* contained in the shell *s*. To calculate a scalar score, we integrate the FSC curve over all frequency shells up to the Nyquist frequency: 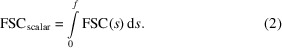
FSC values can range from +1 for perfectly correlated images to 0 for completely uncorrelated images. Negative values (up to −1) imply a negative correlation. An FSC of −1 would represent identical images with opposite contrasts (Penczek, 2020 ▸). In cryo-EM imaging, the FSC_half_ curve between half-dataset maps is used to determine the ‘gold standard’ resolution (Rosenthal & Henderson, 2003 ▸). The frequency at which the FSC curve first falls below a fixed value (usually 0.143) (Rosenthal & Henderson, 2003 ▸) is used as a resolution estimate.

*F*_1_ and *F*_2_ in equation (1) can be represented by a common signal term and an additional noise term, *F*_1_ = *S* + *N*_1_, *F*_2_ = *S* + *N*_2_, where *N*_1_ and *N*_2_ are realizations of noise *N*. With this, FSC_half_ becomes (Rosenthal & Henderson, 2003 ▸) 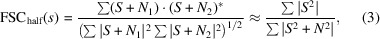
when signal and noise are uncorrelated and data in the half-sets are on the same scale. Using the above notation, we can also write FSC between an ideal map and a map reconstructed from a complete dataset. The ideal map has no noise term, and the noise of the full-dataset map becomes 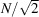 when compared with the half-dataset noise *N*. This so-called *C*_ref_ can be expressed as a function of FSC_half_ [substituting with the result of equation (3), see also Rosenthal & Henderson (2003 ▸)]: 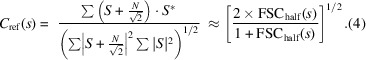
Signal and noise in the above equations are uncorrelated only in a statistical sense (*i.e.* the expectation value of *S* · *N* = 0), and for a given map, noise realization might have a non-zero correlation with the signal (van Heel & Schatz, 2017 ▸, 2005 ▸). Furthermore, equation (4) has many solutions, in the sense that a noise-free map that fulfills equation (4) is not unique (Ramlaul *et al.*, 2019 ▸).

Our loss function is the mean absolute difference between the *C*_ref_ in equation (4) (calculated using readily available FSC_half_ curves) and FSC_FD_, which is calculated between the average of half-maps (used as the network input map for denoising) and the denoised output map:
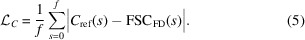
Calculating the loss, we assume that the average of two half-maps represents a map reconstructed from a complete dataset.

The FSC_FD_ between a noise-free map and the average of two half-set maps should completely overlap with *C*_ref_. An FSC_FD_ value above *C*_ref_ indicates that the denoised map still contains some residual noise (under-denoised), while a value below *C*_ref_ points to a loss of signal. The lower the 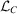, the closer the FSC_FD_ of our network output is to the *C*_ref_, indicating a more effective denoising operation. The loss function 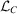 is differentiable since it is directly derived from the FSC function, which itself is differentiable (Kaczmar-Michalska *et al.*, 2022 ▸) and can thus be readily applied in gradient-based model training. The 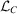 loss allows us to perform Fourier space based model optimization for the real-space theoretical noise-free map, even without actually having noise-free maps to drive the model training. The correlations of signal and noise realizations in the training maps should not limit the loss performance since the training is performed over many maps, and these correlations should average out.

### 2.2. Bias analysis

The denoising process might introduce a spurious bias signal to the map (Palovcak *et al.*, 2020 ▸). For example, the self-supervised *Noise2Void* model introduces checkerboard artefacts to the denoised images (Höck *et al.*, 2023 ▸). In general, bias manifestation can be of any form and can potentially harm the denoised map quality. Can the magnitude of bias be quantified and used to assess denoising model quality?

A noisy map consists of signal and noise, 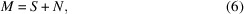
and a denoised map consists of signal, bias and some leftover noise, 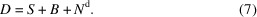
A variance of signal [var(*S*)], noise [var(*N*)], bias [var(*B*)] and leftover noise after denoising [var(*N*^d^)] can be calculated from the noisy and denoised half-maps. We follow derivations provided by Palovcak *et al.* (2020 ▸) to show that these properties are readily calculable starting from covariances of noisy and denoised maps. Elementary relations between variance and covariance of variables are used in the calculations and are provided below. With variables *X*, *Y*, *V* and *Z*: 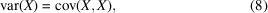

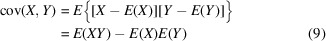
and
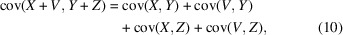
where cov(·, ·) is the covariance and *E*(·) is the expectation value. Equations (8) and (10) imply that
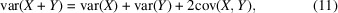
and if *X* and *Y* are independent, *E*(*XY*) = *E*(*X*)*E*(*Y*), equation (9) implies that cov(*X*, *Y*) = 0. Assuming that *S* and *N* are independent, the variance of signal and noise in 3D cryo-EM maps, var(*S*) and var(*N*), can be calculated using the noisy half-maps *M*_1_ and *M*_2_: 
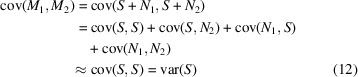
and
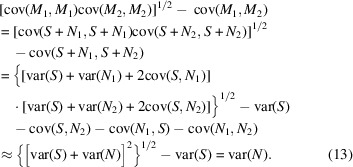
Furthermore, assuming that *B* and *N*, *B* and *N*^d^, and *S* and *N*^d^ are independent, we can calculate the variance of *B* using the noisy (*M*_1_, *M*_2_) and denoised (*D*_1_, *D*_2_) half-maps: 
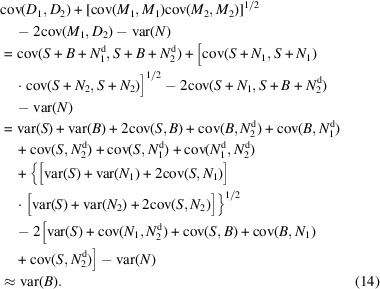
A similar derivation for *N*^d^ is provided below:
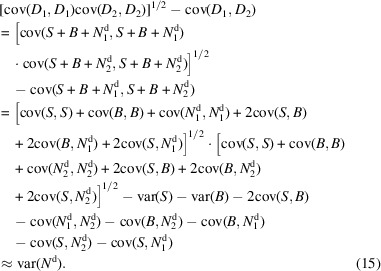
The covariances can be calculated separately for each frequency shell *s* in 3D maps:
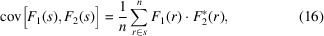
where *n* is the number of voxels *r* in the shell *s*. Usefully, the *Electron Microscopy Data Analytical Toolkit* (*EMDA*) (Warshamanage *et al.*, 2022 ▸) provides routines to calculate 3D map covariances.

### 2.3. Network architecture

We use a 3D U-Net-like (Ronneberger *et al.*, 2015 ▸) model with five levels of depth in the contracting path and corresponding five levels in the expanding path. The overall architecture of *crefDenoiser* is enumerated below:

(i) Input layer. The model takes as input a 3D image with a single channel.

(ii) Contracting path. The contracting path consists of five blocks, each containing a 3D convolutional layer with 16 filters, a Leaky ReLU activation function and a 3D MaxPooling layer.

(iii) Bottleneck. The bottleneck consists of a 3D convolutional layer and a Leaky ReLU activation function. This part of the network is responsible for learning the most abstract features of the input data.

(iv) Expanding path. The expanding path also consists of five blocks, each containing a 3D UpSampling layer, a concatenation operation, two 3D convolutional layers and two Leaky ReLU activation functions. The concatenation operation combines the features learned in the contracting path with the upsampled output, allowing the network to use both local and global features for the reconstruction of the denoised image.

(v) Output layer. The final layer of the network is a 3D convolutional layer with a single filter, which outputs the denoised 3D image.

The total number of parameters in the model is 322 881, all of which are trainable.

Since cryo-EM images inherently capture the intricate 3D structures of macromolecules, this architecture is particularly well suited to the task due to its ability to effectively learn spatial hierarchies and extract features from the 3D data. Although the network is trained on patches of maps (see Section 2.4), it is fully convolutional and can denoise whole maps of any size without any architectural restrictions.

### 2.4. Data preparation

Our model was trained on data collected from the EMDB repository. All cryo-EM entries, with an associated mask and two half-maps attached, were downloaded from the online EMDB FTP server. Any entries with size mismatches between the two half-maps and/or the mask files were pruned. The remaining 3710 records, with resolutions in the range 1.22– 9.9 Å, were used in constructing the training and test datasets.

All the half-map pairs were first independently masked and standardized to have a mean voxel value of 0 and an intensity standard deviation of 1. They were then randomly shuffled (as a pair) and split into patches of size 96 × 96 × 96. Any patches that lay completely outside the masking region were removed. Those remaining were then divided in a 1:9 ratio to construct the test and training datasets. The training set finally contains 55 176 such pairs of half-map patches from 3386 maps, while the test dataset contains 6126 pairs from 324 maps. The model was not tuned on validation data, and we do not distinguish between the validation set and the test set. Trained model performance analysis was performed on 50 random maps selected from the test dataset.

### 2.5. Training process

The training process was conducted using the *Adam* (Kingma & Ba, 2014 ▸) optimizer (β_1_ = 0.9, β_2_ = 0.999, ε = 10^−8^) with an initial learning rate of 0.0003. The learning rate was reduced exponentially with a decay rate of *k* = 0.7 every ten epochs. The model was trained for 195 epochs with a batch size of 6 on three NVIDIA A100 80GB GPUs. The training time was ∼120 h. After each epoch, the model’s performance was evaluated on the validation set, and the model weights were saved. The training convergence analysis is shown in Fig. S2 of the supporting information.

### 2.6. Map sharpening

*C*_ref_-denoised maps may be further sharpened. Here, the selected denoised maps were sharpened only to facilitate graphical comparison with the published maps, and were not used for any quantitative analysis. Local sharpening with *Phenix* software (Adams *et al.*, 2010 ▸), with the resolution threshold set to be slightly lower than the published resolution (−0.5 Å), was chosen. The sharpening method was automatically selected using *phenix.auto_sharpen* (Terwilliger *et al.*, 2018 ▸).

## 3. Results

### 3.1. Comparison with *EMReady*, *LAFTER* and *Topaz*^*Tomo*^ methods

**Figure 1:**
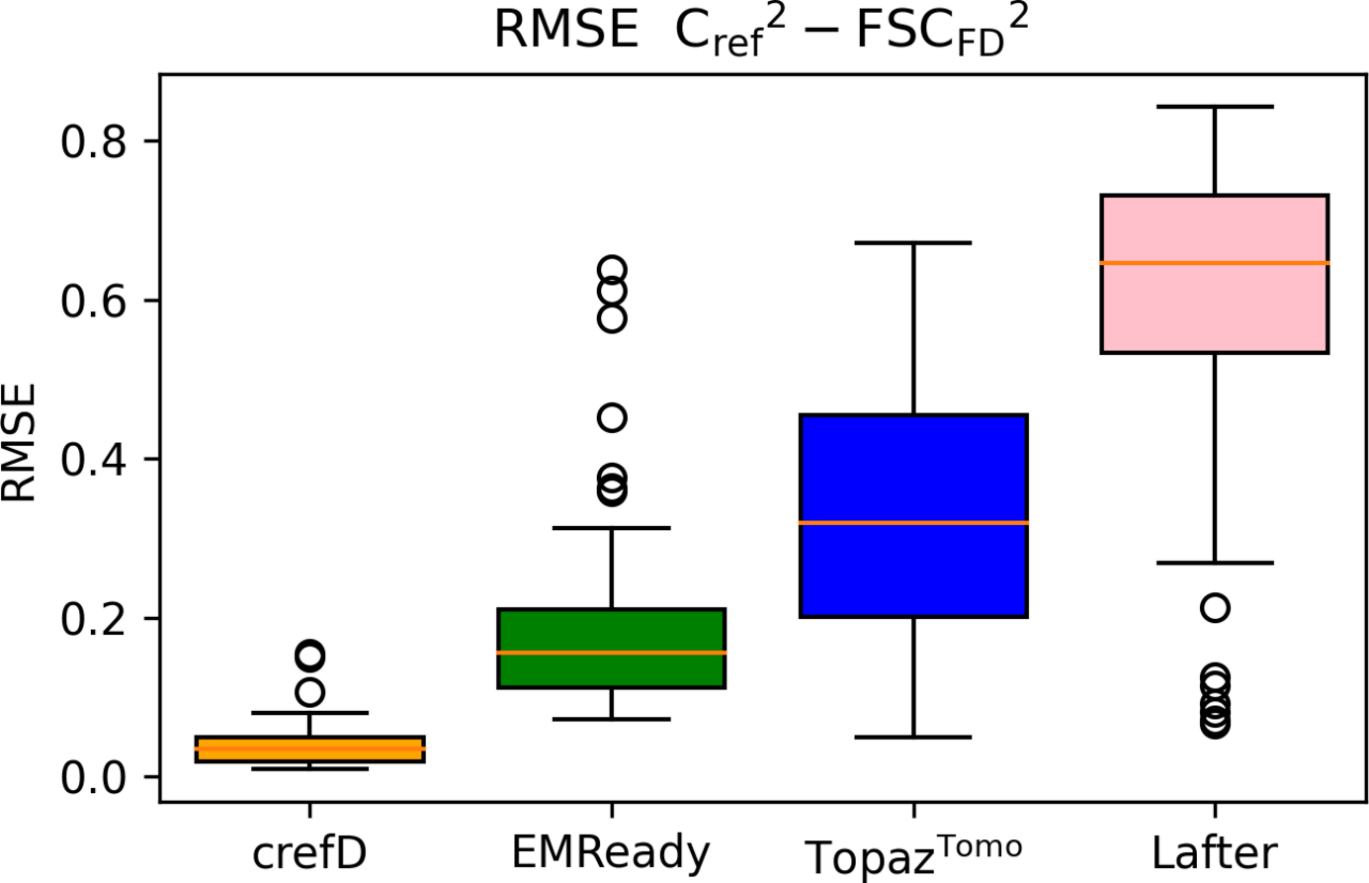
Analysis of denoising performance for a test set of cryo-EM maps. Root mean square error (RMSE) between 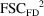 and 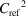 curves is shown for 50 test masked maps processed using *crefDenoiser*, *EMReady*, *Topaz*^*Tomo*^ and *LAFTER* as box and whisker plots. The solid central line depicts the median and the boxes represent the interquartile range. The whiskers span the distribution, excluding any outliers denoted by circles. RMSE is calculated for the squared values to ensure the numerical stability of calculations.

For a random set of masked denoised test maps (*n* = 50), we calculated FSC_FD_ and compared it with the theoretical *C*_ref_, analyzing root mean square difference between the two curves. Fig. 1 ▸ shows results for denoising with *crefDenoiser*, *LAFTER* and *EMReady*, as well as the *Topaz*^*Tomo*^ 3D denoiser (Bepler *et al.*, 2020 ▸). As is evident from the plot, *crefDenoiser* has the smallest value (is the closest to *C*_ref_) of all the methods by a significant margin, which is not surprising since *crefDenoiser* was trained to minimize this map property. *EMReady* performs second best, while *Topaz*^*Tomo*^ and *LAFTER* demonstrate lower performance. In Fig. S1, FSC_FD_ and *C*_ref_ for one representative map, EMD-23276 (Zhang *et al.*, 2021 ▸), are shown. FSC_FD_ for *crefDenoiser* follows closely the theoretical *C*_ref_ curve. The pattern visible for this map repeats in other test maps: *EMReady* shows deviations in FSC_FD_ from *C*_ref_ in lower frequencies (possibly due to the low accuracy of denoising lipids and other molecules not present in the atomistic structures used to construct reference maps for the *EMReady* model training), *LAFTER* shows rather large (as in Fig. S1) or rather low (see outliers in Fig. 1 ▸) RMSE to the *C*_ref_ curve, and *Topaz*^*Tomo*^ under-denoises the high-frequency signal. The RMSE results of the FSC_FD_ to *C*_ref_ curves alone are not enough to claim that the maps are close to the true biological reconstruction, since various denoised maps can minimize the RMSE measure. Further analysis of the denoised map properties is provided below.

*EMReady* (He *et al.*, 2023 ▸) authors use the resolution at which the FSC between a pair of maps falls to one-half (*i.e.* FSC-0.5) as a metric for the model evaluation. Here, we compare the performance of *crefDenoiser* with the other denoisers using this same metric.

**Figure 2:**
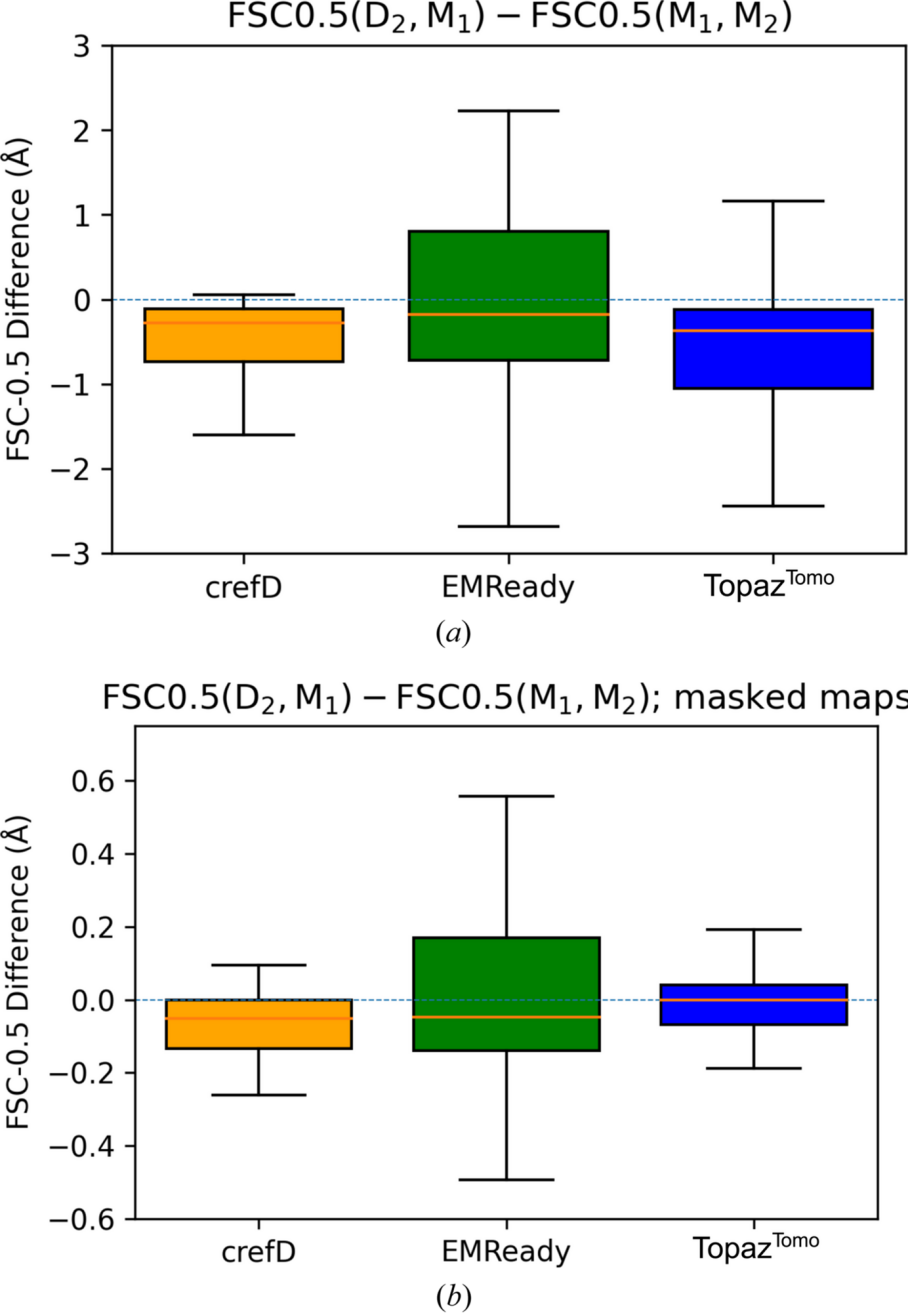
Comparison of FSC-0.5 for maps denoised with the *crefDenoiser*, *EMReady* and *Topaz*^*Tomo*^ methods. The difference in FSC-0.5 values between denoised–noisy half-maps and noisy-to-noisy half-maps (*M*_1_, *M*_2_) for 50 EMDB test entries is shown as box-whisker plots for (*a*) non-masked and (*b*) masked maps. The plots depict distributions of FSC-0.5 difference for each method. The solid central line depicts the median and the boxes represent the interquartile range.

For this we calculated FSC-0.5 values for the noisy [FSC-0.5(*M*_1_, *M*_2_)] and denoised–noisy half-map pairs [FSC-0.5(*D*_2_, *M*_1_)] for the 50 test maps. *LAFTER* is not designed to denoise single half-maps, so it was excluded from this analysis. In Fig. 2 ▸, we present the change of FSC-0.5 after applying *crefDenoiser*, *EMReady* and *Topaz*^*Tomo*^ models for masked and non-masked maps.

For the majority of the analyzed maps, the FSC-0.5 change is negative for all three methods when the analyzed maps are unmasked (with a median change around −0.2 Å). A negative change suggests improved quality of the processed maps since the FSC-0.5 shifts to higher resolution values. When masked maps are used as inputs, *crefDenoiser* and *EMReady* facilitate a negative change, albeit smaller (with a median change of less than −0.1 Å). *Topaz*^*Tomo*^ does not manage to significantly affect the FSC-0.5 with the masked inputs.

The presented analysis suggests that the *crefDenoiser* model can perform half-map denoising, even though it was trained on the full-data density maps. We reason that the noisy maps from the large training set have a broad range of noise levels, and most of the half-maps fall within that range.

**Figure 3:**
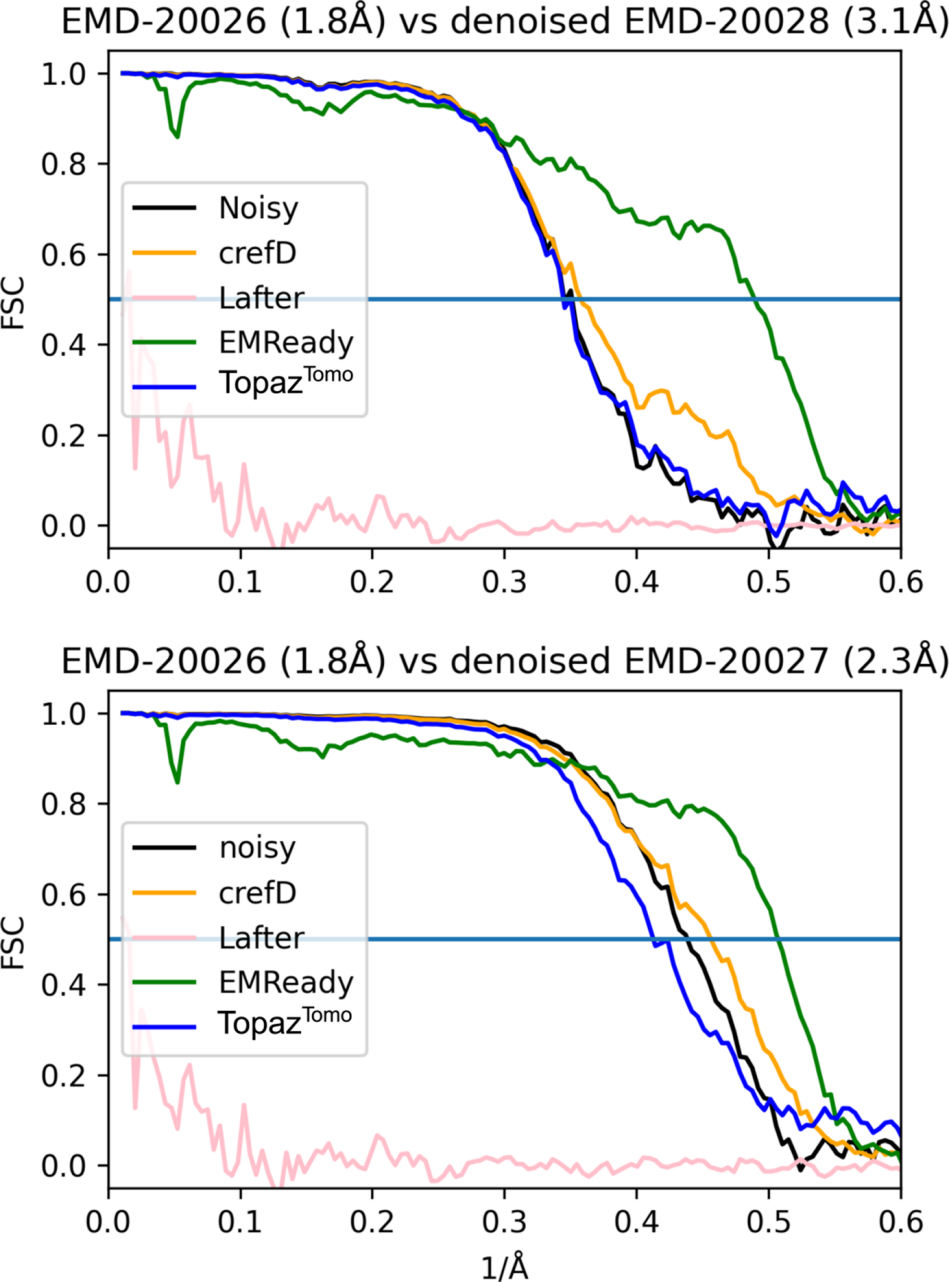
Comparison of denoised maps with a higher-resolution map. Half-maps for human apoferritin EMDB entries EMD-20027 (2.3 Å) and EMD-20028 (3.1 Å) were averaged (within entry), and the mean maps were subsequently denoised. Next, FSC curves to the mean map of the high-resolution entry, EMD-20026 (1.8 Å), were calculated. Maps EMD-20026, EMD-20027 and EMD-20028 were obtained by processing different fractions of the same single-particle dataset (Pintilie *et al.*, 2020 ▸). Denoising with *EMReady* and *crefDenoiser* improves FSC curves to the higher-resolution map for masked maps. The maps are part of the test set.

Next, we tested methods by comparing lower-resolution denoised maps with a high-resolution map. The analyzed apoferritin entries EMD-20026 (1.8 Å), EMD-20027 (2.3 Å) and EMD-20028 (3.1 Å) are reconstructed from the same dataset using different fractions of the acquired single-particle images (Pintilie *et al.*, 2020 ▸). In Fig. 3 ▸, we show that denoising with *EMReady* and *crefDenoiser* improves FSC curves for both lower-resolution models (on masked maps). Mainly, the high-frequency part of the FSC curve is changed; the effect is significantly more pronounced for the *EMReady* model. *Topaz*^*Tomo*^ gives little to no improvement for EMD-20028 and actually degrades map quality for EMD-20027 over most of the frequency range. *LAFTER* performs poorly for both maps. We further analyze whether *crefDenoiser* introduces any spurious densities in appoferritin maps in Fig. S3.

**Figure 4:**
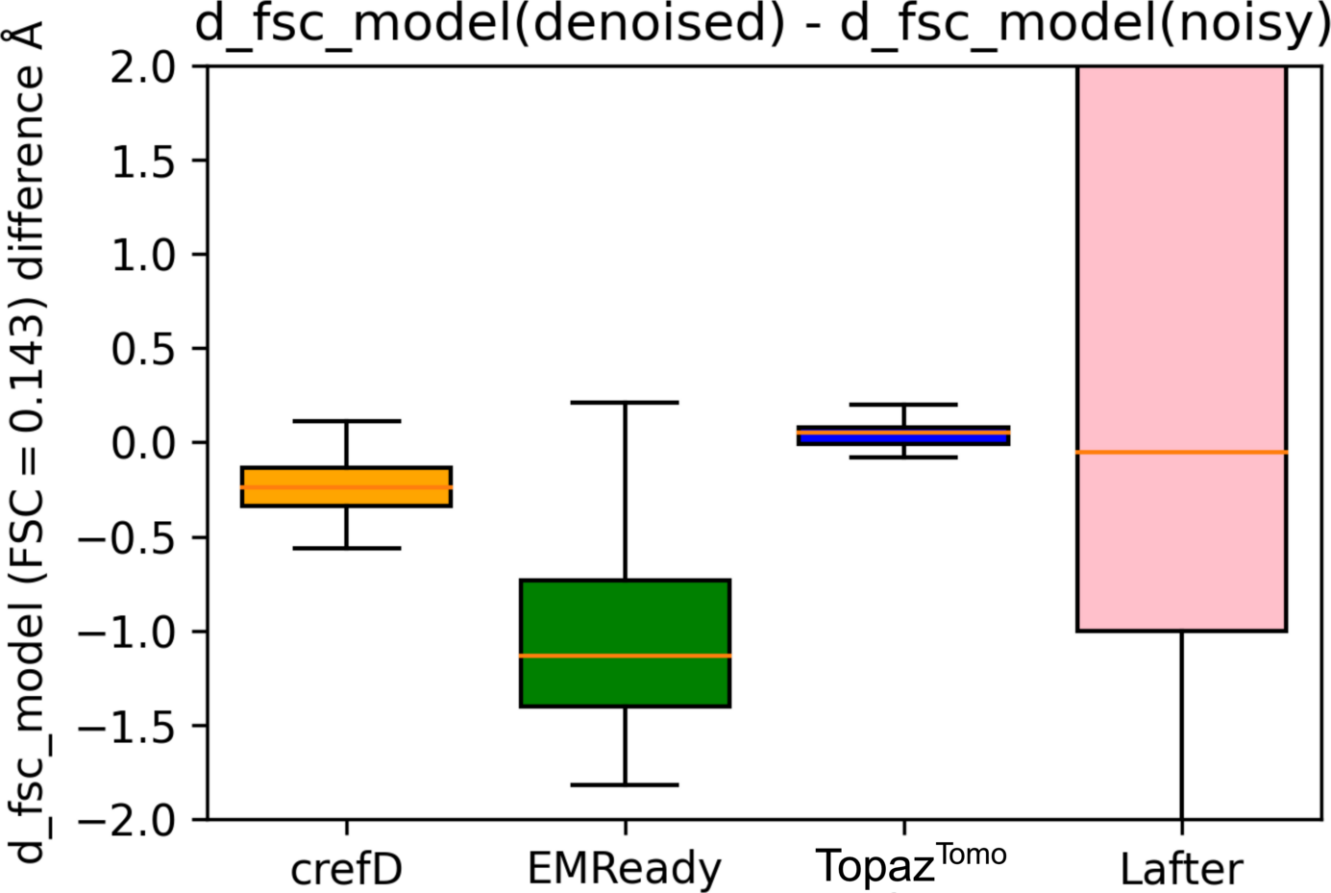
A comparison of the fit of denoised and noisy test-set maps with their published atomic models. The 33 test maps (from 50) for which automatic model extraction was successful were used in the analysis. The masked FSC = 0.143 values were directly computed using *phenix.mtriage* (Adams *et al.*, 2010 ▸).

Unfortunately, the analysis for low-resolution denoised and high-resolution benchmark maps cannot be performed on a larger number of maps due to a lack of accessible data. However, we can approximate this analysis by comparing experimental cryo-EM maps with the maps calculated from atomic models. In Fig. 4 ▸ we demonstrate the difference in fit between 33 full maps from the test set (for which we extracted atomic models in a programmatic way) and their corresponding atomic models, before and after denoising. The fit is evaluated using the d_fsc_model_0143 metric from *phenix.mtriage* (Adams *et al.*, 2010 ▸). This measure signifies the resolution cutoff at which the FSC between the EM map and the atomic model falls below 0.143. We observe that denoising using *crefDenoiser* generates a modest improvement in the resolution cutoff (with a median change around −0.3 Å) while *EMReady* maps demonstrate large similarity with the model maps (with a median change larger than −1 Å). This result is not unexpected since *EMReady* was directly trained using maps simulated from the reference atomic models. On the other hand, *crefDenoiser* was exposed only to the experimental half-maps and still provides maps with improved similarity with the atomic model maps.

For *LAFTER* and *Topaz*^*Tomo*^ processed maps, we do not observe an improved resolution cutoff for most of the test maps. *Topaz*^*Tomo*^ has little to no effect on the fit, while *LAFTER* performs inconsistently and can sometimes degrade the fit by a large amount. For a large fraction of *LAFTER* processed maps, the d_fsc_model_0143 metric could not be calculated.

### 3.2. Comparison with published maps

**Figure 5:**
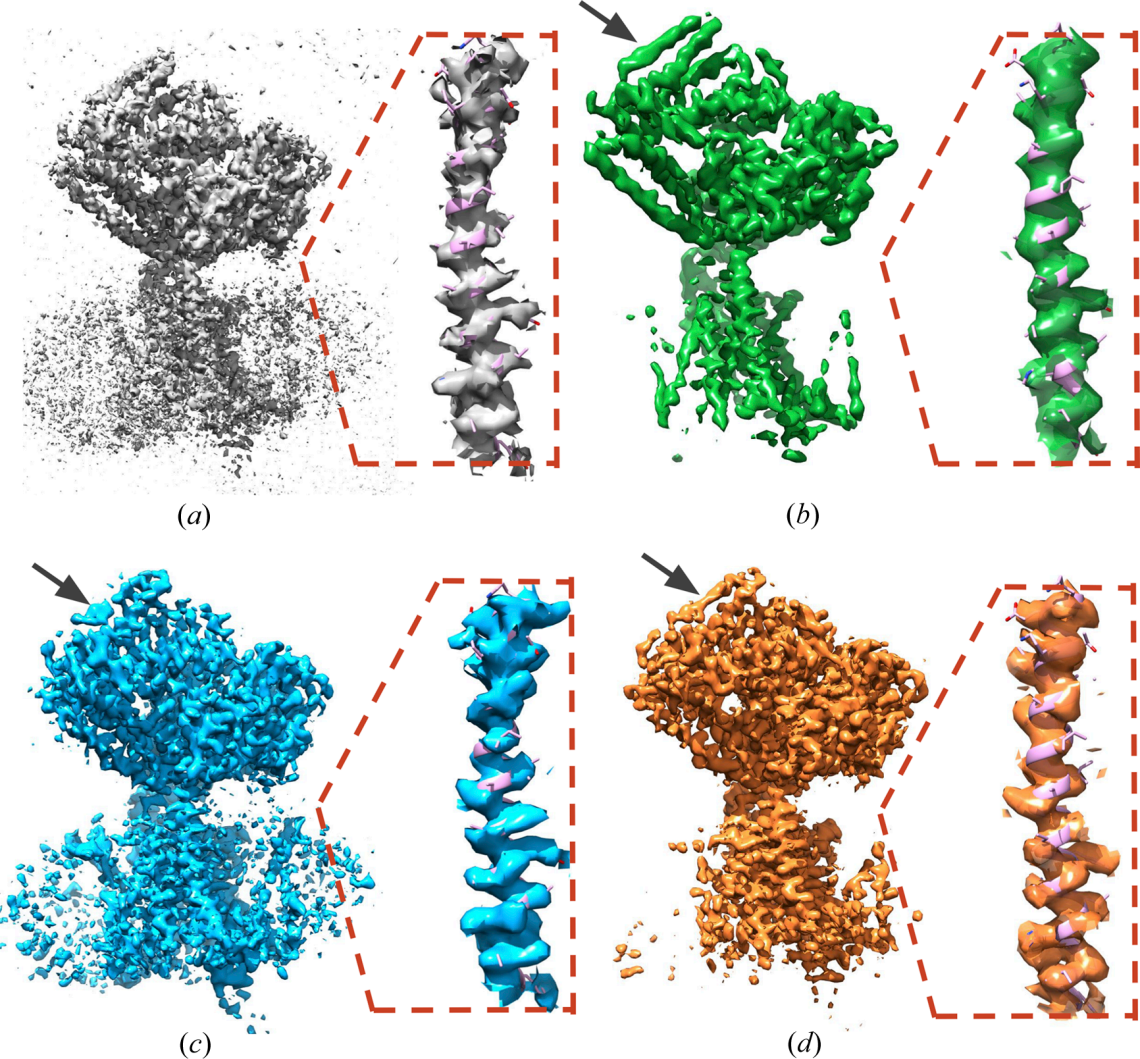
Denoising a medium-resolution map. The map of Sec61 membrane channel from *Saccharomyces cerevisiae* (Itskanov *et al.*, 2021 ▸) and its fragment focused on one selected internal channel helix with the fitted atomistic model are visualized. This map is part of the test set and has a resolution of 4 Å. The noisy map, constructed as a mean of two half-maps, is presented in (*a*). The final published map is shown in (*b*). The noisy sharpened mean map is presented in (*c*). The denoised and sharpened mean map is presented in (*d*). The denoised map preserves lower-resolution motifs (the black-arrow marked α-helix) and high-resolution details (the inset), while the noise is substantially reduced. Contouring was tuned to make the maps most similar.

**Figure 6:**
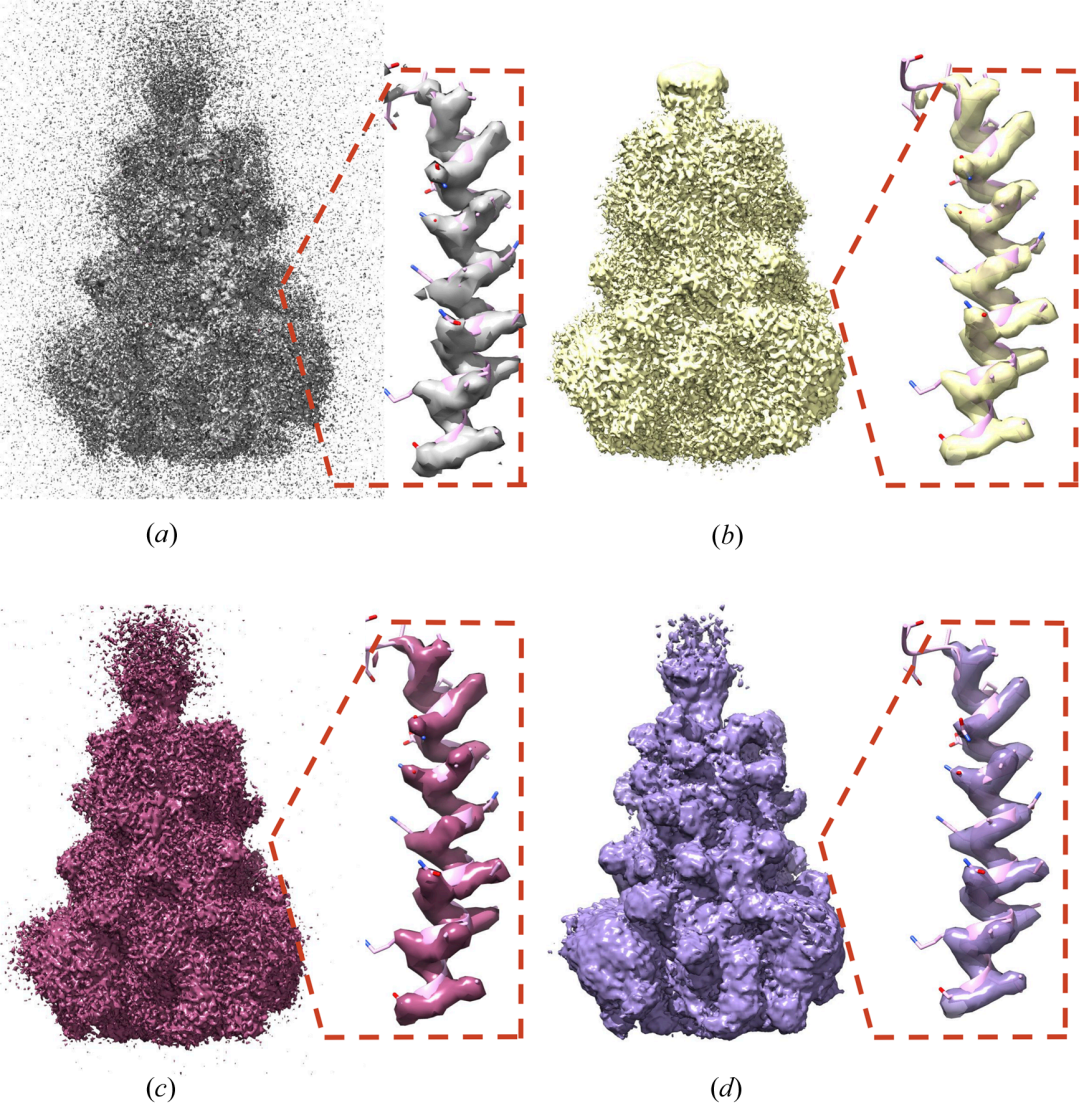
Denoising high-frequency noise. The map of SARS-CoV-1 Spike Protein [EMD-34420 (Zhang *et al.*, 2023 ▸)] and its fragment focused on a single helix with the fitted atomistic model are presented. The map is part of the training set and has a resolution of 2.99 Å. The noisy map, constructed as a mean of two half-maps, is presented in (*a*). The final map published in the EMDB repository is shown in (*b*). The noisy sharpened mean map is presented in (*c*). The denoised and sharpened mean map is presented in (*d*). Contouring was tuned to make the maps most similar. The high-frequency structural features of the published and denoised maps are similar (see the α-helix); however, the denoised map provides a clear outlook of the overall architecture of the spike due to the removal of high-frequency noise.

In Figs. 5 ▸ and 6 ▸, we visually compare *C*_ref_-denoised maps with the published final 3D cryo-EM maps, as deposited by authors in the EMDB repository. For example, in medium-resolution map EMD-22778 (4 Å) of Sec61 membrane channel (Itskanov *et al.*, 2021 ▸), densities of some α-helices improve after denoising (see Fig. 5 ▸), while noise due to lipid densities is mostly removed. To exclude that the effects are only due to sharpening, we also show noisy-sharpened maps. In the case of the SARS-CoV-1 Spike Protein map, EMD-34420 (Zhang *et al.*, 2023 ▸), shown in Fig. 6 ▸, denoising removes high-frequency noise that obscures an overview of the domain positions within the spike map [Fig. 6 ▸(*d*)], while the high-resolution information, in particular densities of amino acid side chains, is unaffected [Figs. 6 ▸(*b*) and 6 ▸(*d*)].

### 3.3. Signal, noise and bias in the denoised maps

**Figure 7:**
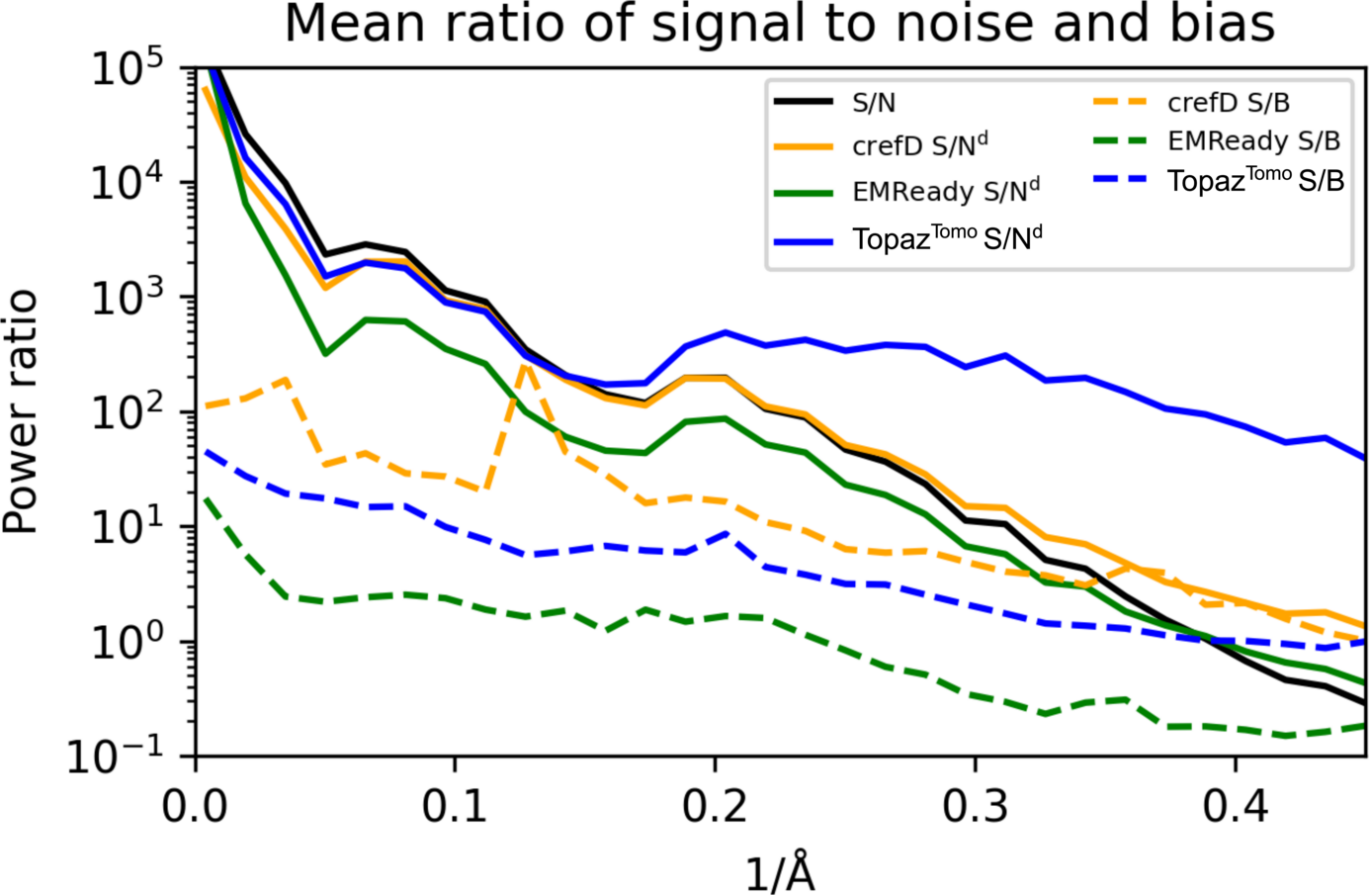
Denoising bias. The ratio of signal to noise [var(*S*)/var(*N*)] and signal to bias [var(*S*)/var(*B*)] is plotted as a function of resolution for noisy and network processed maps. The mean values were calculated with 50 masked maps taken from the test set. Noisy half-maps and denoised half-maps were used to calculate the plotted characteristics, as described in the main text.

In Fig. 7 ▸, we show ratios of signal-to-noise and signal-to-bias powers for the set of 50 test maps. The signal, noise and bias variances are computed as explained in Section 2.2. At first sight, the analysis shows unfavorable signal-to-bias ratios for the high-frequency range of the *EMReady* processed maps. This seems intuitive since the method modifies maps to include additional signal that is digested from the atomic model based ground truth (and both half-maps are modified in a similar manner by *EMReady*). The SNR is similar or worse when compared with noisy maps, suggesting that *EMReady*’s main action is not denoising. In effect, high-frequency noise is (visually) dampened in the *EMReady* processed maps by the much larger bias signal. In the case of the *crefDenoiser*, the SNR is higher in the high-frequency range (denoising effect), while network-based bias is much lower when compared with *EMReady*. However, the overall analysis of data from Fig. 7 ▸ is confusing when the *Topaz*^*Tomo*^ model SNR is considered. The plot suggests very effective denoising of the high-frequency signal by *Topaz*^*Tomo*^, while, with data presented in Fig. S1, we conclude that *Topaz*^*Tomo*^ is a poor denoiser. To better understand the source of this phenomenon, we plot for two maps, signal and noise variances and covariance for two denoised half-maps in Fig. S4. The plot suggests that both noise and signal are dampened by the *Topaz*^*Tomo*^ model in the high-frequency range. Since this effect is correlated, the noise and signal independence assumptions in equations (14) and (15) are not fulfilled, and the variance of noise and bias cannot be accurately computed.

## 4. Discussion and conclusions

This article presents *crefDenoiser*, a self-supervised deep network model for denoising 3D cryo-EM density maps, and compares its performance with the *EMReady*, *LAFTER* and *Topaz*^*Tomo*^ methods. To our knowledge, it is the first network-based model for denoising this type of 3D EM data in a self-supervised manner. The model is trained on ∼3700 experimental maps deposited in the EMDB repository to optimize an ideal noise-free map using the presented theory-based loss. We showcase the benefits of denoising in 3D EM map analysis on real-map examples and provide further data that confirm the improved quality of the denoised maps.

The recent sharpening model *EMReady* (He *et al.*, 2023 ▸) might, as a side effect, also perform map denoising, since it is trained to match simulated maps in which noise is not present. The presented *crefDenoiser*, on the other hand, is trained to maximize the consistency of raw cryo-EM data, *i.e.* half-maps, with the denoised map employing the *C*_ref_ based analytical loss. The presented analysis suggests that *EMReady* enhances maps by ingesting additional signal, which we observe as bias in the analysis presented in Fig. 7 ▸. On the other hand, *crefDenoiser* restores maps by improving the SNR in the high-frequency range but without introducing additional large signal components, *i.e.* map biasing. The map enhancement due to SNR change is rather moderate, while the additional signal in *EMReady* maps moves them closer to higher-resolution maps and to atomic models, as shown in Figs. 3 ▸ and 4 ▸. Still, the observed map enhancement by *crefDenoiser* is not given *per se*. First, the model optimization (minimization of *C*_ref_ based loss) does not guarantee that the model restores the ‘true’ noise-free map because the loss is approximate and the space of solutions is degenerate. It is likely that, similar to models trained on a standard 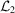 loss [see Menon *et al.* (2020 ▸)], *crefDenoiser* generates a ‘mean’ solution, here a 3D density map, that approximates all possible noise-free maps. Second, *crefDenoiser* is not able to restore the underlying signal if the corruption affects both half-maps similarly; for example, power loss of high-frequency signal in the half-maps.

The two other tested denoising methods, *LAFTER* and *Topaz*^*Tomo*^, do not enhance map quality in our analysis. For *Topaz*^*Tomo*^, quality enhancement was not expected, since the model was trained for cryo electron tomography data. However, analysis of *Topaz*^*Tomo*^-processed maps provides useful insights into the limitations of bias and noise power spectra analysis.

The *C*_ref_ loss is more sensitive to high-frequency signal than a standard 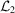 based loss, which seems to be beneficial for model training (Fig. 1 ▸). ‘Noise-to-noise’ models, where one 3D half-map is used as denoising input and the second half-map is used to compute loss, could also be trained with a high-frequency focused loss, such as FSC [equation (1) (Kaczmar-Michalska *et al.*, 2022 ▸), see also Tegunov *et al.* (2021 ▸)], instead of an 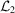 loss. However, the noise-to-noise setup, as used for example in the *M* software (Tegunov *et al.*, 2021 ▸), does not take advantage of both noisy maps during training (one is used to compute loss and the other is used as denoising input). In our model, we use the mean of both half-maps as input for denoising training, with the noise magnitude reduced by 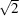. Furthermore, the model is extensively trained on a relatively large dataset (compared with *M*) until convergence, resulting in a versatile denoiser. Training models with a larger training dataset, when it becomes available, might further reduce the leftover noise and improve the denoising results.

While in the presented analysis all the results were calculated for maps that were not part of the training set, it is important to highlight that in real-world applications, the maps requiring denoising could also be incorporated into the training process. This flexibility is made possible by *crefDenoiser*’s ability to train without the need for ground-truth clean maps (self-supervised learning) and has the potential to yield even better denoising results as the model can adapt to more specific noise patterns present in these maps.

We anticipate that the presented model could be beneficial during map analysis and processing steps. Furthermore, since it effectively improves SNR and introduces only low-level bias in the processed maps, it could find applications as a regularizer in 3D map reconstruction pipelines.

## Supplementary Material

Supporting information. DOI: 10.1107/S2052252524005918/fq5024sup1.pdf

## Data Availability

The code and model are available in the Github repository at https://github.com/ajrzepiela/crefDenoiser.
