# Supplementary material for: Refinement of cryo-EM 3D maps with a self-supervised denoising model: crefDenoiser
Source: IUCrJ. 2024 Jul 29;11(Pt 5):821–30. doi: 10.1107/S2052252524005918 (PMC11364040; doi:10.1107/S2052252524005918)
Supplement: Supplementary file 1 [file m-11-00821-sup1.pdf]

IUCrJ

Volume 11 (2024)

Supporting information for article:

Refinement of cryo-EM 3D maps with a self-supervised denoising model: *crefDenoiser*

Ishaant Agarwal, Joanna Kaczmar-Michalska, Simon F. Nørrelykke and Andrzej J. Rzepiela

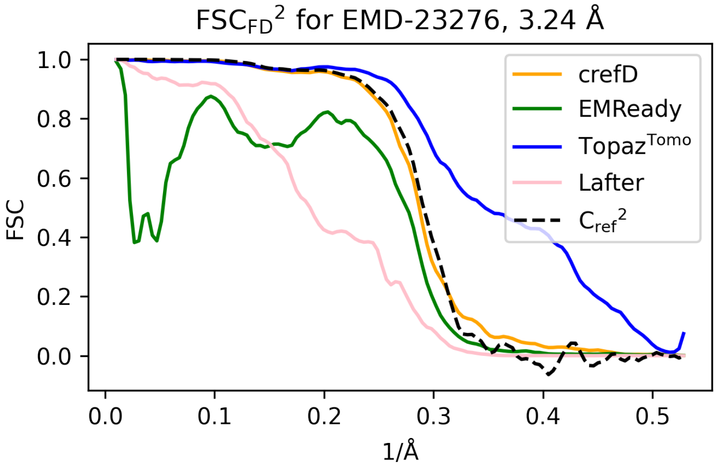

Fig. S1. Analysis of denoising performance for a single cryo-EM masked map, ATP-bound TnsC-TniQ complex (EMD 23276 (Zhang *et al.*, 2021)). FSC_{FD}^2 and C_{ref}^2 are plotted up to the Nyquist resolution.

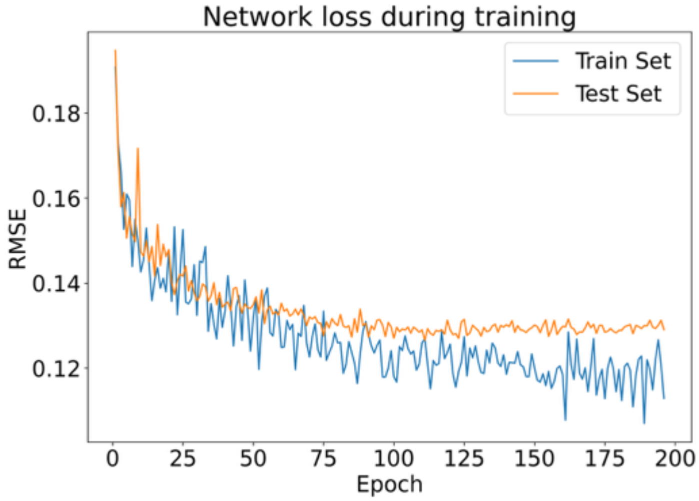

Fig. S2. RMSE between FSC_{FD} and C_{ref} on patches from both test and training sets during training, up to the 195th epoch.

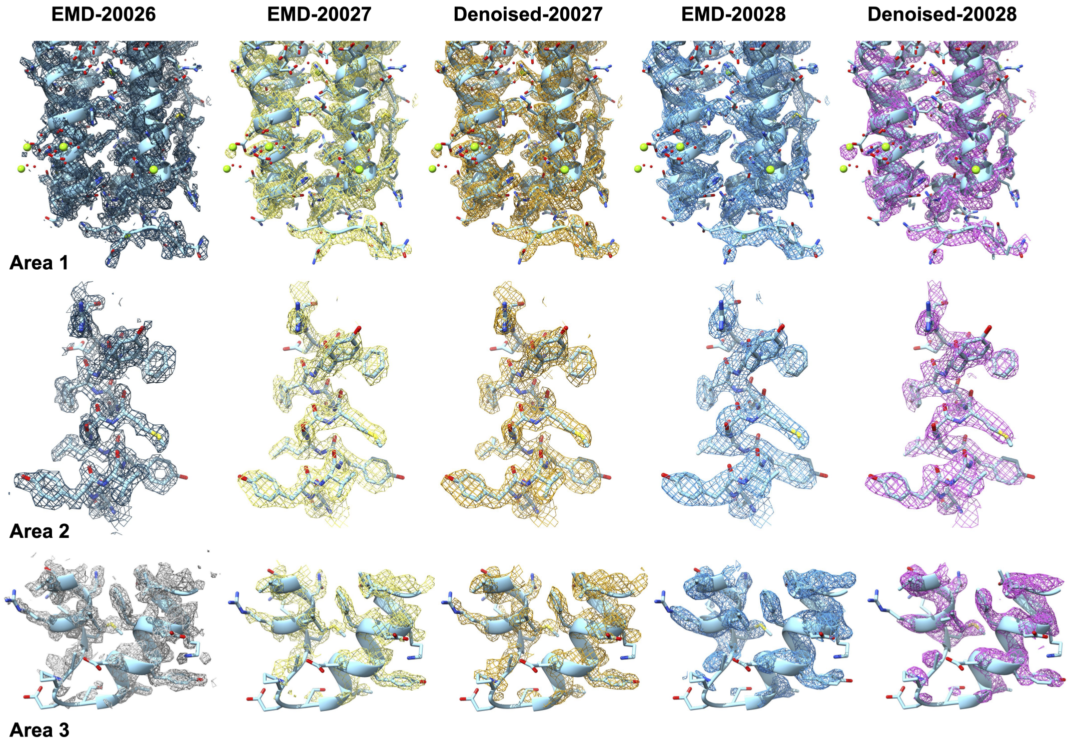

Fig. S3. Denoising does not introduce noticeable false densities to the maps. Comparisons of three areas for the published human apoferritin maps EMD-20026(1.8Å) EMD-20027(2.3Å) and EMD-20028(3.1Å) and two denoised maps (from half maps of sets EMD-20027 and EMD-20028). The denoised maps were sharpened before comparison, as specified in section 2.2. Atomistic model (1.5Å) of apoferritin, 3ajo, is also shown. Contouring is the same for the three areas.

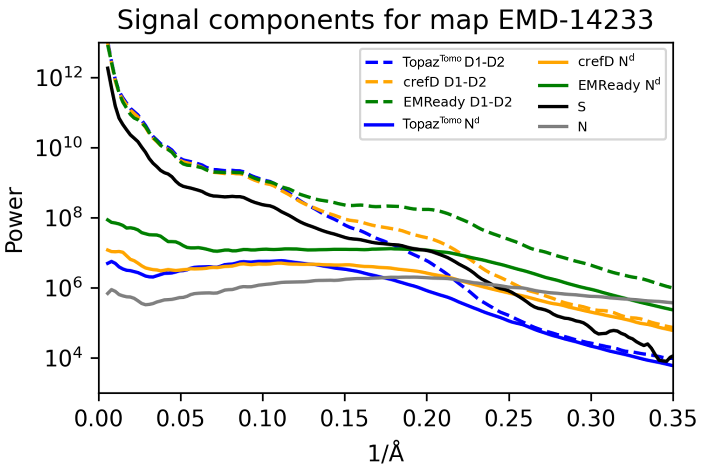

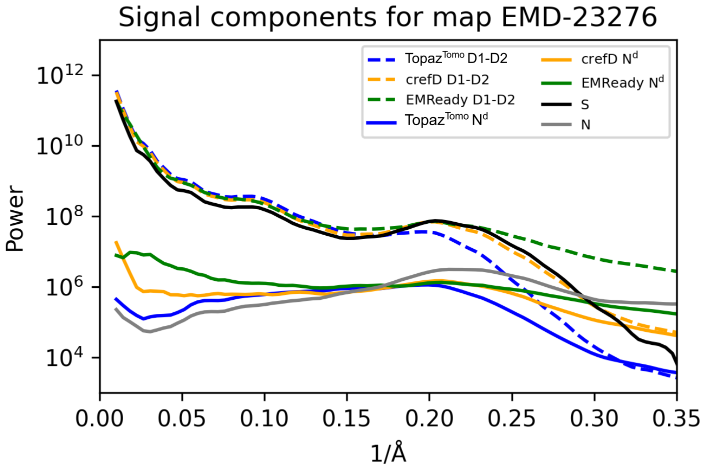

Fig. S4. Signal (S) and noise (N) calculated from noisy maps together with the covariance of denoised maps (D1-D2, depicting S+B) and noise after denoising (N^d) for two test maps (EMD-14233 and EMD-23276). Masking was applied to the maps used in the analysis.
